# Quantitative monitoring of STL with edit distance

**DOI:** 10.1007/s10703-018-0319-x

**Published:** 2018-03-27

**Authors:** Stefan Jakšić, Ezio Bartocci, Radu Grosu, Thang Nguyen, Dejan Ničković

**Affiliations:** 1 Austrian Institute of Technology, Donau-City-Straße 1, Vienna, Austria; 2 Faculty of Informatics, TU Wien, Treitlstraße 3, Vienna, Austria; 3 Infineon Technologies AG, Siemensstraße 2, 9500 Villach, Austria

**Keywords:** Weighted edit distance, Robustness, Hardware monitors, Runtime verification, Dynamic programming

## Abstract

In cyber-physical systems (CPS), physical behaviors are typically controlled by digital hardware. As a consequence, continuous behaviors are discretized by sampling and quantization prior to their processing. Quantifying the similarity between CPS behaviors and their specification is an important ingredient in evaluating correctness and quality of such systems. We propose a novel procedure for measuring robustness between digitized CPS signals and signal temporal logic (STL) specifications. We first equip STL with quantitative semantics based on the *weighted edit distance*, a metric that quantifies both space and time mismatches between digitized CPS behaviors. We then develop a dynamic programming algorithm for computing the robustness degree between digitized signals and STL specifications. In order to promote hardware-based monitors we implemented our approach in FPGA. We evaluated it on automotive benchmarks defined by research community, and also on realistic data obtained from magnetic sensor used in modern cars.

## Introduction

Cyber-physical systems ($\textsc {CPS}$) integrate heterogeneous collaborative components that are interconnected between themselves and their physical environment. They exhibit complex behaviors that often combine discrete and continuous dynamics. The sophistication, complexity and heterogeneity of $\textsc {CPS}$ makes their verification a difficult task. Runtime monitoring addresses this problem by providing a formal, yet scalable, verification method. It achieves both rigor and efficiency by enabling evaluation of systems according to the properties of their individual behaviors.

In the recent past, property-based runtime monitoring of $\textsc {CPS}$ centered around signal temporal logic ($\textsc {STL}$) [29] and its variants have received considerable attention [2, 6, 7, 14, 15, 18, 31]. $\textsc {STL}$ is a formal specification language for describing properties of continuous and hybrid behaviors. In its original form, $\textsc {STL}$ allows to distinguish correct from incorrect behaviors. However, the binary true/false classification may not be sufficient for real-valued behaviors. The classical satisfaction relation can be replaced by a more quantitative *robustness degree* [14, 15, 18] of a behavior with respect to a temporal specification. The robustness degree provides a finer measure of how far is the behavior from satisfying or violating of the specification.

Here we propose a novel quantitative semantics for $\textsc {STL}$ that measures the behavior mismatches in both *space* and *time*. We consider applications in which continuous $\textsc {CPS}$ behaviors are observed by a digital device. In this scenario, continuous behaviors are typically discretized, both in time and space, by an analog-to-digital converter (ADC). As a consequence, we interpret $\textsc {STL}$ over discrete-time digitized behaviors.

We first propose the *weighted* edit distance as an appropriate metric for measuring similarity between $\textsc {CPS}$ behaviors. The weighted edit distance has the following desirable characteristics:

1. It is cumulative, hence it can differentiate between a single and multiple deviations from a reference behavior;
2. It combines spatial and temporal aspects, which are both important when reasoning about CPS behaviors; and
3. It is defined in discrete time, which is an important aspect for the applications that we consider.

We then provide the quantitative semantics for $\textsc {STL}$ based on this distance and discuss the effects of sampling and quantization on the distance value. We develop an efficient online algorithm for computing the robustness degree between a behavior and an $\textsc {STL}$ formula. The algorithm can be directly implemented both in software and hardware. In the former case, the implemented procedure can be connected to the simulation engine of the CPS design and used to monitor its correctness and quality. In the latter case, the resulting implementation can be deployed on the Field Programmable Gate Array (FPGA) and used to monitor real systems or design emulations. We implement the above procedure in Verilog and evaluate it on an automotive benchmark.

We now discuss the main contributions of this work. In contrast to the previous research on STL robustness, we adopt a sampled-time automata-based approach. This choice has several important consequences. First, it allows direct and uniform implementation of STL robustness monitors in both software and hardware and naturally enables monitoring in *real-time*. We implement the algorithms in Verilog and deploy them on FPGAs, thus providing an effective bridge from design-time (e.g. Simulink) to deployment-time (e.g. autonomous vehicles) quantitative monitoring. Second, the automata-based approach is capable of capturing contradictions and tautologies (by checking automata emptiness and universality) and it guarantees that two semantically-equivalent but syntactically-different specifications have the same robustness degree with respect to all behaviors. Finally, we use the weighted edit distance (WED) to reason about the similarity between behaviors and specifications that is novel in the context of STL robustness. This paper is an extended version of [25]. In this paper we extend our preliminary work with new results:

- we provide extensive proofs of the theoretical results in [25]
- we test our approach on an industrial case study with data taken from a real magnetic sensor and verify timing requirements of Single Edge Nibble Transmission Protocol [24], which are crucial for the integrity of information transferred
- we further benchmark our approach with fault-tolerant fuel control system [5] model, taken from the automotive domain

*Organisation of the paper* In Sect. 2 we present the related work while Sect. 3 provides all the necessary formal background. In Sect. 4 we introduce the notion of weighted edit distance. In Sect. 5 we propose a novel approach for computing, using the weighted edit distance, the robustness degree of a discrete signal with respect to an STL property. In Sect. 6 we describe the implementation of our quantitative monitors and we demonstrate our approach on two case studies. Finally, we draw our conclusions in Sect. 7.

## Related work

The Levenshtein (edit) distance [28] has been extensively used in information theory, computer science and bioinformatics for many applications, including approximate string matching, spell checking and fuzzy string searching. Levenshtein automata [37] were introduced to reason about the edit distance from a reference string. A Levenshtein automaton of degree *n* for a string *w* recognizes the set of all words whose edit distance from *w* is at most *n*. A dynamic programming procedure for computing the edit distance between a string and a regular language has been proposed in [42]. The problem of computing the smallest edit distance between any pair of distinct strings in a regular language has been studied in [26]. In contrast to our work, these classical approaches to edit distance consider only operations with simple weights on unordered alphabets and are not applied to dynamic reactive behaviors.

The edit distance for weighted automata was studied in [30], where the authors propose a procedure for computing the edit distance between weighted transducers. A space efficient algorithm for computing the edit distance between a string and a weighted automaton over a tropical semiring was developed in [3]. The resulting approach is generic and allows for instance to assign an arbitrary cost to each substitution pair. However, all substitution pairs must be enumerated by separate transitions. In contrast, we consider signals with naturally ordered alphabets as input strings and hence can efficiently handle substitution over large alphabets by treating allowed input values with symbolic constraints. In addition, we use the edit distance to define the semantics of a temporal specification formalism.

The weighted Hamming and edit distances between behaviors are also proposed in [36], where the authors use it to develop procedures for reasoning about the Lipshitz-robustness of Mealy machines and string transducers. The notion of robustness is different from ours, and in contrast to our work it is not computed against a specification.

The quantitative semantics for temporal logics were first proposed in [18, 35], with the focus on the *spatial* similarity of behaviors, given by their point-wise comparison. The spatial quantitative semantics is sensitive to phase shifts and temporal inaccuracies in behaviors—a small temporal shift in the behavior may result in a large robustness degree change. This problem was addressed in [15], in which $\textsc {STL}$ with spatial quantitative semantics is extended with time robustness. In [2], the authors propose another approach of combining space and time robustness, by extending $\textsc {STL}$ with *averaged* temporal operators. Another approach to determining robustness of hybrid systems using self-validated arithmetics is shown in [19]. Monitoring of different quantitative semantics is implemented in tools such as S-TaLiRo [4] and Breach [13].

The problem of online monitoring robustness was studied more recently in [9, 12]. The authors of [12] propose an online monitoring approach that uses a predictor, which requires for the future fragment of the logic the access to a model of the system. This is in contrast to our black-box view of monitoring. In [9], the authors propose an interval-based approach of online evaluation that allows estimating the minimum and the maximum robustness with respect to both the observed prefix and unobserved suffix of the trace. In our work, we do not provide such estimation about the future. Instead, our robustness value at every point in time gives the distance of the observed prefix from the satisfaction/violation of the specification.

The recent results on using Skorokhod metric [39] to compute the distance between piecewise-linear or piecewise-constant continuous behaviors [10] partially inspired our work. Skorokhod metric quantifies both space and time mismatches between continuous behaviors by allowing application of time distortions in behaviors in order to minimize their point-wise distance. The distortion of the timeline is achieved by applying a retiming function—a continuous bijective strictly increasing function from time domain to time domain. Given a behavior *x*(*t*), the resulting retimed behavior *r*(*x*(*t*)) preserves the values and their order but not the duration between two values. This information-preserving distance relies on continuous time and is not applicable to the discrete time domain—stretching and compressing the discrete time axis results inevitably in an information loss. Finally, the computation of the Skorokhod distance was extended to the flow-pipes in [11] and to the epsilon-tubes in [8],where the authors consider computing the distance between hybrid (continuous and discrete-time) signals. We are not aware of any work addressing the problem of computing the Skorokhod distance between a behavior and a temporal specification.

Our work is also related with the notions of $(\upvarepsilon , \uptau )$-closeness in [1] and $(\upvarepsilon , \uptau )$-similarity (requires the retiming to be order-preserving) introduced in [34] to compare two mixed-analog signals and in conformance testing [1]. The parameters $\uptau$ and $\upvarepsilon$ are used to specify how much it is allowed to wiggle in both time and space in order to transform one trace into another. The main difference with this work is that our distance provides a cumulative measure, while the other notions try to find the max possible discrepancy.

Recently published industrial case study [38] shows an application of STL monitoring for verifying the sensor which uses SENT [24] protocol. We regard that work as completely orthogonal to this paper. The case study focuses on qualitative monitors able to recover upon violation detection and that are able to detect and collect multiple violations in one go. The framework in that paper is limited to a particular class of asynchronous communication protocols. In contrast, this paper is about quantitative monitoring for arbitrary STL properties.

## Preliminaries

In this section, we provide the necessary definitions to develop the algorithm presented in subsequent sections of the paper. We first shortly recall the notion of metric spaces and distances. We then define signals and signal temporal logic. Finally, we introduce a variant of symbolic and weighted symbolic automata.

### Metric spaces and distances

A metric space is a set for which distances between all elements in the set are defined.

#### Definition 1

(*Metric space and distance*) Suppose that ${\mathcal {M}}$ is a set and $d~:~{\mathcal {M}}\times {\mathcal {M}} \rightarrow {\mathbb {R}}$ is a function that maps pairs of elements in ${\mathcal {M}}$ into the real numbers. Then ${\mathcal {M}}$ is a *metric space* with the *distance measure* *d*, if (1) $d(m_{1},m_{2}) \ge 0$ for all $m_{1},m_{2}$ in ${\mathcal {M}}$; (2) $d(m_{1},m_{2}) = 0$ if and only if $m_{1} = m_{2}$; (3) $d(m_{1},m_{2}) = d(m_{2},m_{1})$ for all $m_{1},m_{2}$ in ${\mathcal {M}}$; and (4) $d(m_{1},m_{2}) \le d(m_{1},m) + d(m,m_{2})$ for all $m,m_{1},m_{2}$ in ${\mathcal {M}}$.

Given $m \in {\mathcal {M}}$ and $M \subseteq {\mathcal {M}}$, we can lift the above definition to reason about the distance between an element *m* of ${\mathcal {M}}$ and the subset *M* of ${\mathcal {M}}$ as follows

$$\begin{aligned} d(m,M) = \inf _{m' \in M} d(m,m') \end{aligned}$$

We define the *robustness degree* $\uprho (m,M)$ of *m* with respect to the set *M* as follows

$$\begin{aligned} \uprho (m,M) = \left\{ \begin{array}{ll} d(m,{\mathcal {M}} \backslash M) &{} \text {if } m \in M \\ -d(m,M) &{} \text {otherwise} \end{array} \right. \end{aligned}$$

### Signals

Let *X* be a finite set of variables defined over some domain ${\mathbb {D}}$. Then, a *signal* *s* is a function $s~:~{\mathbb {T}} \times X \rightarrow {\mathbb {D}}$, where ${\mathbb {T}}$ is the time domain^1^. We distinguish between *analog*, *discrete* and *digital* signals. Analog signals have continuous value and time domains. The time domain of discrete signals is the set of integers, while digital signals have in addition their value domain restricted to a finite set. Digital signals can be obtained by *sampling* and *quantization* of analog signals. The conversion of analog to digital signals is at the core of the signal processing field and is in practice done by an *analog-to-digital converter* (ADC).

Sampling is the process of reducing the continuous time in analog signals to the discrete time in the resulting discrete signal. The ideal theoretical sampling function periodically measures the value of the analog signal every *T* time units, where *T* denotes the *sampling interval*. Similarly, we denote by *f* the *sampling frequency*, that is the average number of measurements obtained by sampling in one second, where $f = 1/T$. Given an analog signal $s_a~:~{\mathbb {R}}_{\ge 0} \times X \rightarrow {\mathbb {R}}^{n}$ and a sampling interval *T*, applying the ideal sampling function to $s_a$ results in a discrete signal $s_{disc }~:~{\mathbb {N}} \times X \rightarrow {\mathbb {R}}$ such that $s_{disc }(i, x) = s_a(iT, x)$ for all $i \ge 0$ and $x \in X$.

When sampling real-valued signals, it is impossible to maintain the arbitrary precision of its values, which consequently must be restricted to a finite set. Quantization consists of converting real values to their discrete numerical approximations, and thus allows to map discrete to digital signals. We consider the basic uniform quantization function with a *quantization step* ${\mathsf {Q}}$ which is defined as follows

$$\begin{aligned} Q(r) = {\mathsf {Q}}\cdot \lfloor |r|/{\mathsf {Q}}+ 0.5 \rfloor , \end{aligned}$$

where $r \in {\mathbb {R}}$. We note that the quantization can be decomposed into two stages, *classification* and *reconstruction*. The classification function *c* maps the real input value into an integer index *k*, and the reconstruction function *y* converts *k* into the actual discrete approximation of the input. Hence, we have that $Q(r) = y(c(r))$ where

$$\begin{aligned} \begin{array}{lcl} c(r) &{} = &{} \lfloor |r|/{\mathsf {Q}}+ 0.5 \rfloor \\ y(k) &{} = &{} {\mathsf {Q}}\cdot k \\ \end{array} \end{aligned}$$

The decomposition of the quantization into two independent stages has a practical advantage—without loss of generality, we can from now directly work with digital signals obtained after the classification stage with their value domain being a finite subset of ${\mathbb {N}}$. We also restrict ourselves to signals that have *finite-length* and hence are of the form $s_{dig }~:~[0,l) \times X \rightarrow [v_{min}, v_{max}]$, where [0, *l*) and $[v_{min},v_{max}]$ are intervals in ${\mathbb {N}}$, and *X* is now the set of variables defined over the domain $[v_{min}, v_{max}]$. We extend the signal notation *s*(*i*, *X*) to denote the vector ${\mathbb {D}}^{|X|}$ of all variable values in *X* at time *i*. From now on, we refer to digital signals of finite length simply as signals and denote them by *s*.

### Signal temporal logic

In this paper, we study signal temporal logic ($\textsc {STL}$) with both *past* and *future* operators interpreted over digital signals of finite length.^2^

Let *X* be a finite set of variables defined over a finite interval domain ${\mathbb {D}} = [v_{min },v_{max }] \subseteq {\mathbb {N}}$. We assume that *X* is a metric space equipped with a distance *d*. The syntax of an $\textsc {STL}$ formula $\upvarphi$ over *X* is defined by the grammarwhere $x \in X$, $\sim \in \{ <, \le \}$, $u \in {\mathbb {D}}$, *I* is of the form [*a*, *b*] or $[a, \infty )$ such that $a,b \in {\mathbb {N}}$ and $0 \le a \le b$. The other standard operators are derived as follows: $\textsf {true}= p \vee \lnot p$, $\textsf {false}= \lnot \textsf {true}$, $\upvarphi _1 \wedge \upvarphi _2 = \lnot (\lnot \upvarphi _1 \vee \lnot \upvarphi _2)$, , , , ,  and .

The semantics of an $\textsc {STL}$ formula with respect to a signal *s* of length *l* is described via the satisfiability relation $(s,i) \models \upvarphi$, indicating that the signal *s* satisfies $\upvarphi$ at the time index *i*, according to the following definition where ${\mathbb {T}} = [0, l)$.We note that we use the semantics for  and  that is strict in both arguments and that we allow punctual modalities due to the discrete time semantics. Given an $\textsc {STL}$ formula $\upvarphi$, we denote by $L(\upvarphi )$ the *language* of $\upvarphi$, which is the set of all signals *s* such that $(s,0) \models \upvarphi$.

### Automata and weighted automata

In this section, we define a variant of *symbolic automata* [41] and also present its *weighted* extension. The notion of weighted automata and its well-established theory is provided in [16] while symbolic weighted automata accepting input string over not necessarily finite set have been investigated in [21].

Similarly to the definition of STL, we consider ${\mathbb {D}}= [v_{min },v_{max }]$ to be the finite interval of integers equipped with the distance *d* and let *X* to be a finite set of variables defined over ${\mathbb {D}}$. The variable valuation *v*(*x*) is a function $v~:~X \rightarrow {\mathbb {D}}$, which we naturally extend to the valuation *v*(*X*) of the set *X*. A variable *constraint* $\upgamma$ over *X* is defined by the grammar in negation normal form $\upgamma := x \le c~|~\lnot (x \le c)~|~\upgamma _{1} \vee \upgamma _{2}~|~\upgamma _{1} \wedge \upgamma _{2}$, where $x \in X$ and $c \in {\mathbb {D}}$. We denote by $\Gamma (X)$ the set of all constraints definable over *X*. Given the valuation *v*(*X*) and a constraint $\upgamma$ over *X*, we write $v(X) \models \upgamma$ when *v*(*X*) satisfies $\upgamma$.

#### Definition 2

(*Symbolic automata*) We define a *symbolic automaton* ${\mathcal {A}}$ as the tuple ${\mathcal {A}}= ({\mathbb {D}}, X, Q, I, F, \Delta )$, where ${\mathbb {D}}$ is the finite *alphabet*, *X* is a finite set of variables over ${\mathbb {D}}$, *Q* is a finite set of *states*, $I \subseteq Q$ is the set of *initial states*, $F \subseteq Q$ is the set of *final states* and $\Delta = \Delta _{X} \cup \Delta _{\upvarepsilon }$ is the *transition relation*, where $\Delta _{X} \subseteq Q \times \Gamma (X) \times Q$ and $\Delta _{\upvarepsilon } \subseteq Q \times \{ \upvarepsilon \} \times Q$ are sets of transitions that consume an input letter and *silent* transitions, respectively.

Given a $q \in Q$, let ${\mathcal {E}}(q)$ denote the set of states reachable from *q* by following $\upvarepsilon$-transitions in $\Delta$ only. Formally, we say that $p \in {\mathcal {E}}(q)$ iff there exists a sequence of states $q_{1}, \ldots , q_{k}$ such that $q = q_{1}$, $(q_{i}, \upvarepsilon , q_{i+1}) \in \Delta$ for all $0 \le i < k$, and $p = q_{k}$. Let $s~:~[0,l) \times X \rightarrow {\mathbb {D}}$ be a signal. We say that *s* is a *trace* of ${\mathcal {A}}$ if there exists a sequence of states $q_{0}, \ldots , q_{l}$ in *Q* such that $q_{0} \in {\mathcal {E}}(q)$ for some $q \in I$, for all $0 \le i < l$, there exists $(q_{i}, \upgamma , q_{i+1}) \in \Delta$ for some $\upgamma$ such that $s(i, X) \models \upgamma$ and $q_{i+1} \in {\mathcal {E}}(q)$ and $q_{l} \in F$. We denote by $L({\mathcal {A}})$ the set of all traces of ${\mathcal {A}}$. A *path* $\uppi$ in ${\mathcal {A}}$ is a sequence  such that $q_{0} \in I$ and for all $0 \le i < n$, $\updelta _{i}$ is either of the form $(q_{i}, \upgamma , q_{i+1})$ or $(q_{i}, \upvarepsilon , q_{i+1})$. We say that $\uppi$ is *accepting* if $q_{n} \in F$. Given a trace $s~:~[0,l) \times X \rightarrow {\mathbb {D}}$ and a path $\uppi = q_{0} \cdot \updelta _{0} \cdot q_{1} \cdot \updelta _{1} \cdots \updelta _{n-1} \cdot q_{n}$, we say that *s* induces $\uppi$ in ${\mathcal {A}}$ if $\uppi$ is an accepting path in ${\mathcal {A}}$ and its projection to observable alphabet letters gives *s*. We denote by $\Pi ({\mathcal {A}},s) = \{ \uppi ~|~s \text { induces } \uppi \text { in } {\mathcal {A}}\}$ the set of all paths in ${\mathcal {A}}$ induced by *s*.

We now introduce *weighted* symbolic automata, by adding a weight function to the transitions of the symbolic automaton, relative to the consumed input letter.

#### Definition 3

(*Weighted symbolic automata*) A *weighted symbolic automaton* ${\mathcal {W}}$ is the tuple ${\mathcal {W}}= ({\mathbb {D}}, X, Q, I, F, \Delta , \uplambda )$, where ${\mathcal {A}}= ({\mathbb {D}}, X, Q, I, F, \Delta )$ is a symbolic automaton and $\uplambda ~:~ \Delta \times ({\mathbb {D}}^{|X|} \cup \{ \upvarepsilon \}) \rightarrow {\mathbb {Q}}^{+}$ is the weight function.

Let *s* be a signal of size *l* and $\uppi = q_{0} \cdot \updelta _{0} \cdots \updelta _{n-1} \cdot q_{n}$ a path in ${\mathcal {W}}$ induced by *s*. The value of $\uppi$ in ${\mathcal {W}}$ subject to *s*, denoted by $v_{ \uppi } (s, {\mathcal {W}})$, is the sum of weights associated to the transitions in the path $\uppi$ and subject to the signal *s*. We define the *value* $v(s,{\mathcal {W}})$ of *s* as the minimum value from all the paths in ${\mathcal {W}}$ induced by *s*, i.e. $v(s,{\mathcal {W}}) = \min _{\uppi \in \Pi ({\mathcal {W}},s)} v_{ \uppi } (s, {\mathcal {W}})$.

## Weighted edit distance

Measuring the similarity of sequences is important in many application areas, such as information theory, spell checking and bioinformatics. The *Hamming distance* $d_{H}$ is the most basic and common string measure arising from the information theory. It measures the minimum number of *substitution* operations needed to match equal length sequences. The *edit distance* $d_{E}$ extends the Hamming distance with two additional operations, *insertion* and *deletion* and is defined as the minimum accumulation of edit operation costs used to transform one sequence into the other.

Neither of these metrics provide satisfactory solution for comparing digitized signals. They are defined over unordered alphabets and associate fixed costs to different kinds of operations. In contrast, the value domain of digital signals admits a natural notion of a distance representing the difference between two signal valuations. In addition, the Hamming distance provides only point-wise comparisons between sequences and consequently does not account for potential timing discrepancies in the sampled signals. Two discrete signals that differ only in a constant time delay will typically have a large Hamming distance. The edit distance addresses this problem by allowing us to bridge the time shifts using insertion and deletion operations.

Inspired by [30, 36], we propose the *weighted edit distance* as the measure for comparing the similarity of two discrete signals. It adopts the insertion and deletion operations from the edit distance and adapts the substitution operation to the ordered alphabets. Since we consider multi-dimensional signals, we extend the cost of the substitution operation to take into account different variable valuations.

Let *X* be a finite set of variables defined over some interval domain ${\mathbb {D}}= [v_{min },v_{max }]$. Given two valuation vectors $a,b \in {\mathbb {D}}^{|X|}$ of *X*, we denote by $d_{M}(a, b)$ the *Manhattan distance* [27] between *a* and *b*, where $d_{M}(a,b) = \Sigma _{i=0}^{|X|-1} |a_{i} - b_{i}|$. Let $w_{i},w_{d} \in {\mathbb {Q}}$ be weight constants for the insertion and deletion operations. We then define the *costs* of the substitution $c_{s}$, insertion $c_{i}$ and deletion $c_{d}$ operations as follows: $(1) c_{s}(a,b) = d_{M}(a,b); (2) c_{i} = w_{i}; (3) c_{d} = w_{d}$. The definition of the WED adapts the classical edit distance recursive definition with the new costs.

### Definition 4

(*Weighted edit distance*) Let $s_{1}~:~[0,l) \times X \rightarrow {\mathbb {D}}$ and $s_{2}~:~[0,l) \times X \rightarrow {\mathbb {D}}$ be discrete-time signals. The *weighted edit distance* $d_{W}(s_{1},s_{2})$ equals to $d_{l,l}(s_{1},s_{2}):$


### Proposition 1

The weighted edit distance is a distance.

*Remark* We chose the Manhattan distance for the substitution cost because it combines the absolute difference of several signal components.

We now further motivate the use of the weighted edit distance and discuss in more depth its characteristics. We do this by comparing the weighted edit distance ($d_{W}$) to the Hamming distance ($d_{H}$) and to the distance based on the infinity norm ($d_{max }$). In order to compare these three distances, we record the data from a device implementing an automotive communication protocol. We manually manipulate the data to illustrate specific distance properties. We note that we normalize the two cumulative distances with the total number of data samples, in order to have comparable results.

**Fig. 1:**
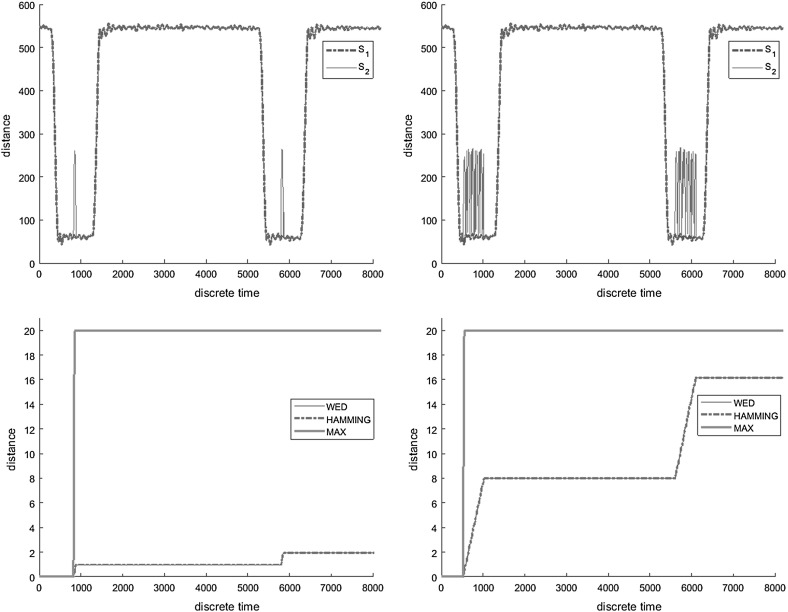
Measuring similarity $d_{i,i}(s_1,s_2)$ between a reference $s_1$ and a measured $s_2$ behavior—single versus multiple deviations

We first study the cumulative property of WED. Figure 1a, b depict two scenarios, both consisting of a reference ($s_{r}$) and a measured ($s_{r}$) behavior. In the first scenario, the two behaviors are equivalent, except for a short spike that happens during each pulse. In the second scenario, the spikes are continuously repeated. Figure 1c, d show the evolution of the three distances over time, where the distance value at time *t* corresponds to the distance between the reference and measured behavior prefixes of size *t*. We can observe that $d_{max }$ measures the maximum deviation between $s_{r}$ and $s_{m}$ and hence does not distinguish between a single and multiple deviations. On the other hand, both $d_{H}$ and $d_{W}$ are cumulative, and the distance between the reference and the measured behavior increases with the number of deviations.

**Fig. 2:**
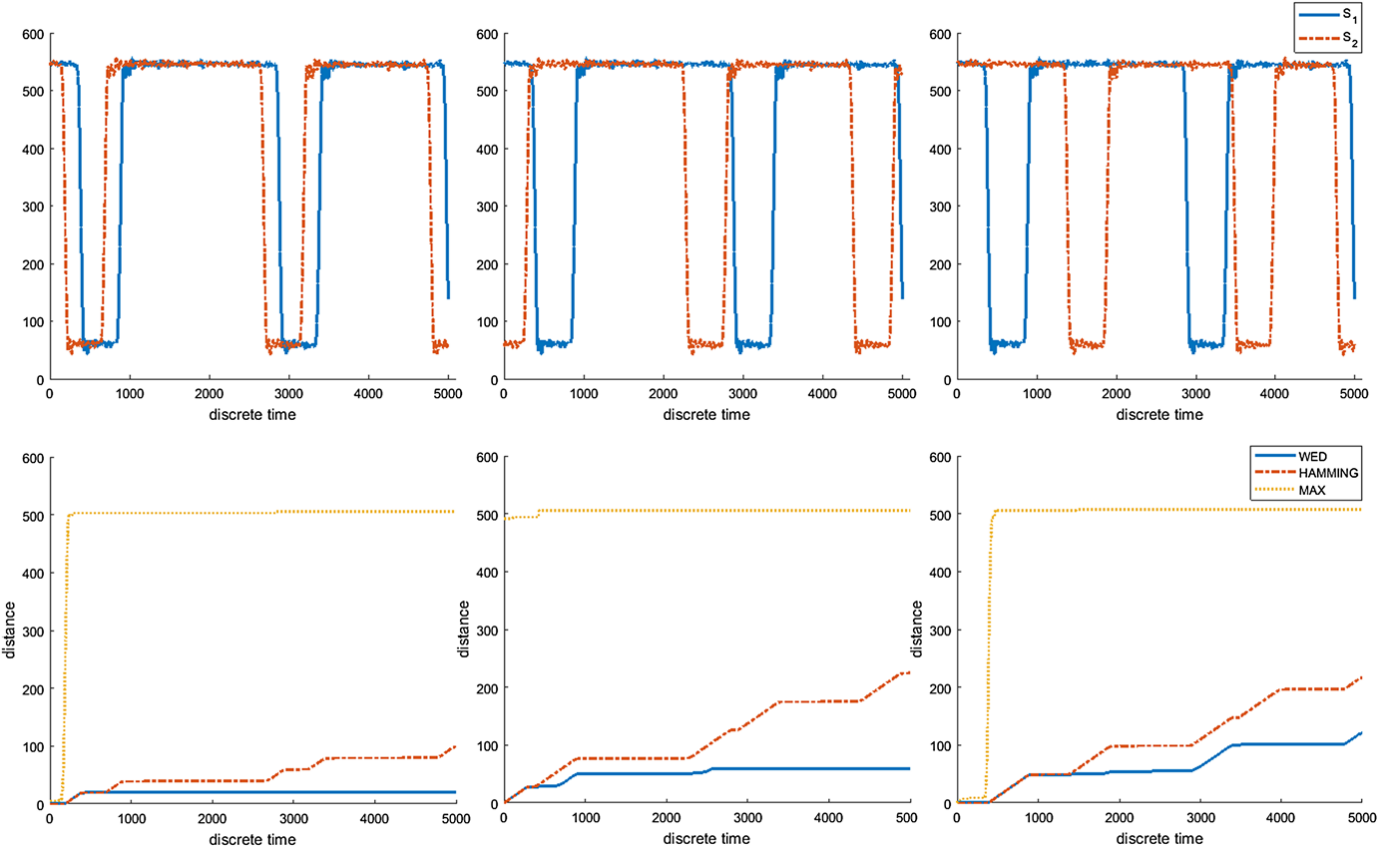
Measuring similarity $d_{i,i}(s_1,s_2)$ between a reference $s_1$ and a measured $s_2$ behavior—phase shifts

Figure 2a–c show three scenarios with the measured signal being equivalent to the reference signal shifted by an increasing amount, respectively. Figure 2d–f show the evolution of $d_{W}$, $d_{H}$ and $d_{max }$ over time. We first note that $d_{max }$ does not make the distinction between the three scenarios. Second, we can observe that $d_{W}$ grows slower than $d_{H}$ over time. This happens because $d_{W}$ counts a small number of insertion and deletion operations, while $d_{H}$ accumulates all the pointwise differences between $s_r$ and $s_m$ over time. Finally, we notice that $d_{H}$ does not make a distinction between the second and the third scenario, despite the different time shifts. This happens because in both scenarios the pulses from the measured signal are superimposed over the the non-pulse segments of the reference behavior. In contrast, $d_{W}$ makes a distinction between the two situations and assigns a higher distance to the third scenario.

**Fig. 3:**
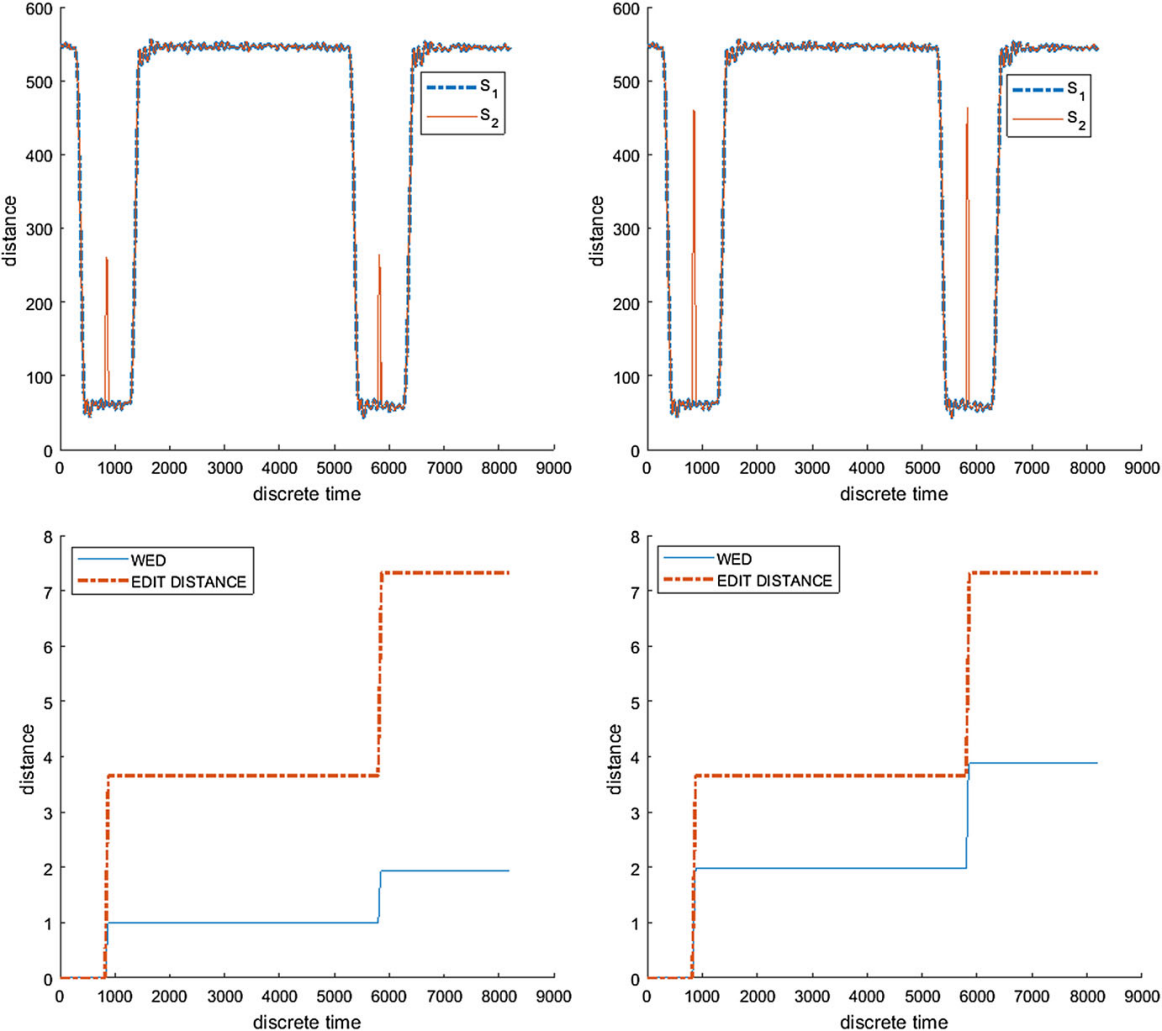
Measuring similarity $d_{i,i}(s_1,s_2)$ between a reference $s_1$ and a measured $s_2$ behavior—magnitude of deviations

Finally, we illustrate the difference between the weighted edit distance and the classical edit distance ($d_{E}$). Figure 3a, b show two scenarios consisting of a reference and a measured behavior, that are in both cases the same, except for a short spike in each pulse. In the first scenario, the magnitude of the spike is smaller than in the second scenario. Figure 3c, d depict the evolution of $d_{W}$ and $d_{E}$ over time. We can see that in contrast to $d_{W}$, $d_{E}$ cannot distinguish between the two scenarios because its substitution operation has a fixed cost.

### Sampling, quantization and weighted edit distance

We compute the WED between digital signals resulting from physical behavior observations after sampling and quantization. In this section, we discuss the effect of inaccuracies introduced by these operations on the WED.

Let *s* be an analog signal, *T* a sampling period and ${\mathsf {Q}}$ a quantization step. We assume that *s* has a band limit $f_{M}$ and $T \le 1/(2f_{M})$. We denote by *s*[*T*] the discrete signal obtained from *s* by sampling with the period *T*, and by $s[T][{\mathsf {Q}}]$ the digital signal obtained from *s*[*T*] by quantization with the step ${\mathsf {Q}}$.

**Fig. 4:**
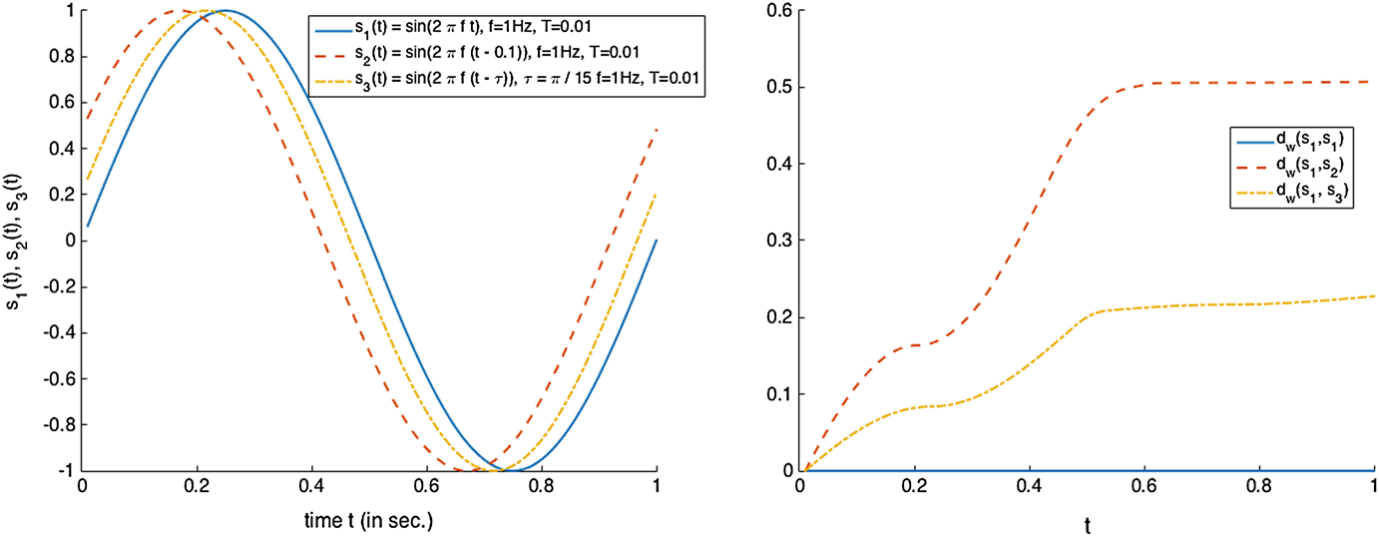
Weighted edit distances $d_{W}(s_{1},s_{2})$ and $d_{W}(s_{1},s_{3})$, where $s_{1}(t)= \sin (2 \uppi f t)$, $s_{2}(t) = \sin (2 \uppi f (t- 0.1))$, $s_{3}(t) = \sin (2 \uppi f (t - \uptau ))$, $T = 0.01$, $f = 1Hz$ and $\uptau = \uppi /15$

We cannot directly relate the WED to the analog signals, because it is not defined in continuous time. However, this distance allows tackling phase shifts in the sampled signals. Consider two analog signals $s_{1}(t)$ and $s_{2}(t - \uptau )$ such that $\uptau = iT$ for some $i \ge 0$ and their sampled variants $s_{1}[T](t)$ and $s_{2}[T](t)$. It is clear that with $2\cdot i$ insertion and deletion operations, $s_{2}[T]$ can be transformed into $s_{1}[T]$ such that their remaining substitution cost equals to 0. This situation is illustrated in Fig. 4 (see signals $s_{1}$ and $s_{2}$). We see that the distance between the two signals initially grows due to the insertion and deletion operations, but that eventually it becomes perfectly stable.

Now consider another signal $s_{3}(t) = s_{1}(t - \uptau )$ such that $\uptau$ is not a multiple of *T*. In this case, the sampled signal $s_{3}[T](t)$ cannot be perfectly transformed into $s_{1}[T](t)$ by using insertion and deletion operations because of the mismatch between the sampling period and the phase shift. As a consequence, the distance between $s_{1}[T](t)$ and $s_{3}[T](t)$ will accumulate substitution costs due to this mismatch. This scenario is also depicted in Fig. 4 (see signals $s_{1}$ and $s_{3}$). The figure shows that after an initial steep increase of the distance due to the insertion and deletion operations, its value does not converge, but continues slowly increasing due to the accumulation of remaining substitution costs.

It is obvious that the actual distance between two behaviors is affected by the sampling frequency. We refer to [40] for the survey on the sampling theory, a field that studies the effects of sampling continuous behaviors.

### Normalized weighted edit distance

The weighted edit distance is an accumulative distance. It follows that the distance between two behaviors depends on several factors, including: 1) the size of the value domains; 2) the frequency at which the two signals are sampled; and 3) the total duration of the trace. For instance, the comparison of two analog behaviors sampled at two different frequencies can result in completely different absolute distance values. In order to have a more uniform robustness valuation that is less affected by the above factors, we propose *normalizing* the robustness values as follows.

Given signals $s_{1}, s_{2}$ of length *l* defined over *X*, the value domain ${\mathbb {D}}= [v_{min }, v_{max }]$, we define the normalized weighted edit distance, which is always bounded by [0, 1] as follows:

$$\begin{aligned} d^{\#}_{W}(s_{1}, s_{2}) = \frac{d_{W}(s_{1},s_{2})}{l|X|(v_{max }-v_{min })}. \end{aligned}$$

## Weighted edit robustness for signal temporal logic

In this section, we propose a novel procedure for computing the *robustness degree* of a discrete signal with respect to an $\textsc {STL}$ property. In our approach, we set $c_{i}$ and $c_{d}$ to be equal to $|X|(v_{max }- v_{min })$. In other words, the deletion and insertion costs are equal to the largest substitution cost. The rationale behind this choice is that by inserting/deleting a data point, we can add/remove the maximum value from the domain in the worst case.

### From STL to weighted edit automata

**Fig. 5:**
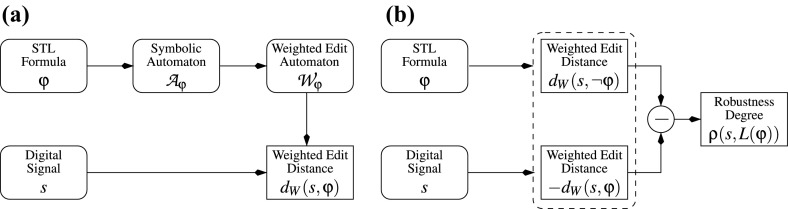
Computation of **a** $d_{W}(s,\upvarphi )$ and $\uprho (s,\upvarphi )$

Our procedure relies on computing the WED between a signal and a set of signals, defined by the specification. It consists of several steps, illustrated in Fig. 5. We first translate the $\textsc {STL}$ formula $\upvarphi$ into a symbolic automaton ${\mathcal {A}}_{\upvarphi }$ that accepts the same language as the specification. The automaton ${\mathcal {A}}_{\upvarphi }$ treats timing constraints from the formula enumeratively, but keeps symbolic guards on data variables^3^. We then transform ${\mathcal {A}}_{\upvarphi }$ into a *weighted edit automaton* ${\mathcal {W}}_{\upvarphi }$, a weighted symbolic automaton that accepts all the signals but with the value that corresponds to the WED between the signal and the specification (Fig. 5a). We propose an algorithm for computing this distance. Computing the robustness degree between a signal and an STL specification follows from the calculation of their WED, as shown in Fig. 5b.

Let *X* be a set of finite variables defined over the domain ${\mathbb {D}}= [v_{min },v_{max }] \subseteq {\mathbb {N}}$. We consider an STL formula $\upvarphi$ defined over *X*. Let $s~:~[0,l) \times X \rightarrow {\mathbb {D}}$ be a digital signal.

#### From $\upvarphi$ to ${\mathcal {A}}_{\upvarphi }$

In the first step, we translate the STL specification $\upvarphi$ into the automaton ${\mathcal {A}}_{\upvarphi }$ such that $L(\upvarphi ) = L({\mathcal {A}}_{\upvarphi })$. The translation from $\textsc {STL}$ interpreted over discrete time and finite valued domains to finite automata is standard, and can be achieved by using for instance on-the-fly tableau construction [20] or the temporal testers approach [33]. We note that we need to accommodate these classic constructions to the finitary semantics of the temporal logic by adapting accordingly the acceptance conditions (see for instance [17] for the interpretation of LTL over finite traces).

##### Example 1

**Fig. 6:**
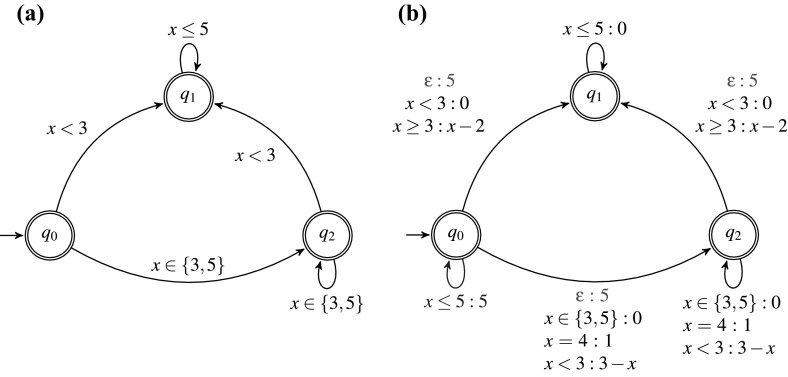
**a** ${\mathcal {A}}_{\upvarphi }$ accepting $L(\upvarphi )$—all states are accepting and **b** ${\mathcal {W}}_{\upvarphi }$

Consider the past $\textsc {STL}$ formula , where *x* is defined over the domain [0, 5]. The resulting automaton ${\mathcal {A}}_{\upvarphi }$ is shown in Fig. 6a.

#### From ${\mathcal {A}}_{\upvarphi }$ to ${\mathcal {W}}_{\upvarphi }$

In this step, we translate the automaton ${\mathcal {A}}_{\upvarphi }$ to the weighted edit automaton ${\mathcal {W}}_{\upvarphi }$. The automaton ${\mathcal {W}}_{\upvarphi }$ reads an input signal and mimics the weighted edit operations. In essence, ${\mathcal {W}}_{\upvarphi }$ accepts every signal along multiple paths. Each accepting path induced by the signal corresponds to a sequence of weighted edit operations needed to transform the input signal into another one allowed by the specification. The value of the least expensive path corresponds to the weighted edit distance between the input signal and the specification. The weighted automaton ${\mathcal {W}}_{\upvarphi }$ explicitly treats substitution, insertion and deletion operations, by augmenting ${\mathcal {A}}_{\upvarphi }$ with additional transitions and associating to them the appropriate weight function. We now provide details of the translation and describe the handling of weighted edit operations. Let ${\mathcal {A}}_{\upvarphi } = ({\mathbb {D}}, X, Q, I, F, \Delta )$ be the symbolic automaton accepting the language of the specification $\upvarphi$.

*Substitution* In order to address substitutions in the automaton, we define a new set of *substitution* transitions $\Delta _{s}$ and associate to them the weight function $\uplambda _{s}$ as follows. Given $q,q' \in Q$, let $\upgamma (q,q') = \bigvee _{(q,\upgamma ,q') \in \Delta } \upgamma$. Then, we have:

- $(q, \textsf {true}, q') \in \Delta _{s}$ if there exists $(q, \upgamma , q') \in \Delta$ for some $\upgamma$; and
- $$\uplambda _{s}((q,\textsf {true},q'),v) = d_{M}(v, \upgamma (q,q'))$$, for all $$v \in {\mathbb {D}}^{|X|}$$.

We define the Manhattan distance of valuation *v* from a $\upgamma (q,q')$ as the minimum of Manhattan distances of the valuation *v* from all the possible valuations that satisfy $\upgamma (q,q')$: $d_{M}(v, \upgamma (q,q')) = min \{ d_{M}(v,w)\}, \forall w \in W$ where $W = \{ w ~|~ w \models \upgamma (q,q') \}$.

Intuitively, we replace all the transitions in ${\mathcal {A}}_{\upvarphi }$ with new ones that have the same source and target states. We relax the guards in the new transitions and make them enabled for *any* input. On the other hand, we control the cost of making a transition with the weight function $\uplambda _{s}$, which computes the substitution cost needed to take the transition with a specific input. This cost is the Manhattan distance between the input value and the guard associated to the original transition.

*Deletion* Addressing deletion operations consists in adding self-loop transitions that consume all the input letters to all the states with the deletion cost $c_{d} = |X|(v_{max }- v_{min })$, thus mimicking deletion operations. We skip adding a self-loop transition to states that already have the same substitution self-loop transition—according to our definition $c_{d} \ge c_s(a,X)$ for all *a*, hence taking the deletion transition instead of the substitution one can never improve the value of a path and is therefore redundant. We define the set of deletion transitions $\Delta _{d}$ and the associated weight function $\uplambda _{d}$ as follows:

- $$(q, \textsf {true}, q) \in \Delta _{d}$$ if $$(q, \textsf {true}, q) \not \in \Delta _{s}$$; and
- $\uplambda _{d}(\updelta , v) = c_{d}$ for all $\updelta \in \Delta _{d}$ and $v \in {\mathbb {D}}^{|X|}$.

*Insertion* In order to mimic the insertion operations, we augment the transitions relation of ${\mathcal {W}}_{\upvarphi }$ with silent transitions. For every original transition in $\Delta$, we associate another transition with the same source and target states, but labeled with $\upvarepsilon$ and having the insertion cost $c_{i} = |X|(v_{max } - v_{min })$. Formally, we define the set of insertion transitions $\Delta _{i}$ and the associated weight function $\uplambda _{i}$ as follows:

- $(q, \upvarepsilon , q') \in \Delta _{i}$ if $(q, \upgamma , q') \in \Delta$ for some $\upgamma$; and
- $$\uplambda _{i}(\updelta , \{\upvarepsilon \}) = c_{i}$$ for all $$\updelta \in \Delta _{i}$$.

Given the symbolic automaton ${\mathcal {A}}_{\upvarphi } = ({\mathbb {D}}, X, Q, I, F, \Delta )$ accepting the language is the tuple $({\mathbb {D}}, X, Q, I, F, \Delta ', \uplambda ')$, where $\Delta ' = \Delta _{s} \cup \Delta _{d} \cup \Delta _{i}$ and $\uplambda '(\updelta , v) = \uplambda _{s}(\updelta ,v)$ if $\updelta \in \Delta _{s}$, $\uplambda '(\updelta , v) = \uplambda _{d}(\updelta ,v)$ if $\updelta \in \Delta _{d}$ and $\uplambda '(\updelta , \upvarepsilon ) = \uplambda _{i}(\updelta ,\upvarepsilon )$ if $\updelta \in \Delta _{i}$.

##### Example 2

The weighted edit automaton ${\mathcal {W}}_{\upvarphi }$ obtained from ${\mathcal {A}}_{\upvarphi }$ is illustrated in Fig. 6b. Both automatons from Fig. 6b use the same input alphabet ${\mathbb {D}}= \{ 0,1,2,3,4,5 \}$. The blue transitions, such as (*A*, 0, *A*) with weight 5, correspond to the deletion transitions. The red transitions, such as $(A,\upvarepsilon , B)$, correspond to the insertion transitions.

The resulting weighted automaton ${\mathcal {W}}_{\upvarphi }$ allows determining the weighted edit distance between a signal *w* and the formula $\upvarphi$, by computing the value of *s* in ${\mathcal {W}}_{\upvarphi }$.

##### Theorem 1

$$d_{W}(s,\upvarphi ) = v(s, {\mathcal {W}}_{\upvarphi })$$.

The consequence of this Theorem is that two symbolic automata that accept the same language will always give the same distance from the same input.

### Computing the value of a signal in a weighted edit automaton

We now present an on-the-fly algorithm Val, shown in Algorithm 1, that computes the value of a signal *s* in a weighted automaton ${\mathcal {W}}$. In every step *i*, the algorithm computes the minimum cost of reaching the state *q* with the prefix of *s* consisting of its first *i* values. After reading a prefix of *s*, we may reach a state $q \in Q$ in different ways with different costs. Note that it is sufficient to keep the state with the minimum value in each iteration. It follows that the algorithm requires book keeping |*Q*| state value fields in every iteration. We now explain the details of the algorithm. The procedure first initializes the costs of all the states in ${\mathcal {W}}$ (see Algorithm 2). The initial states are set to 0 and the non-initial ones to $\infty$. Then, we compute the effect of taking the $\upvarepsilon$ transitions without reading any signal value. It is sufficient to iterate this step |*Q*| times, since within |*Q*| iterations, one is guaranteed to reach a state *q* that was already visited with a smaller value *v*. In every subsequent iteration *i*, we first update the state values by applying the cost of taking all transitions labeled by *s*(*i*, *X*) and then update the effect of taking $\upvarepsilon$ transitions |*Q*| times. The weight function of a substitution cost is computed as follows: $\uplambda (v,x \le k)$ gives 0 if $v \le k$, and $v-k$ otherwise; $\uplambda (v,\lnot (x \le k))$ is symmetric; $\uplambda (v, \upvarphi _{1} \wedge \upvarphi _{2}) = \max (\uplambda (v,\upvarphi _{1}), \uplambda (v, \upvarphi _{2}))$ and $\uplambda (v, \upvarphi _{1} \vee \upvarphi _{2}) = \min (\uplambda (v,\upvarphi _{1}), \uplambda (v, \upvarphi _{2}))$.

Upon termination, the algorithm returns the minimum cost of reaching an accepting state in the automaton.

#### Theorem 2

Val$$(s, {\mathcal {W}}) = v(s, {\mathcal {W}})$$.

#### Theorem 3

Given a signal *s* of length *l* defined over *X* and a weighted automaton ${\mathcal {W}}$ with *n* states and *m* transitions, $\textsf {Val}(s,{\mathcal {W}})$ takes in the order of ${\mathcal {O}}(lnm))$ iterations to compute the value of *s* in ${\mathcal {W}}$, and requires in the order of ${\mathcal {O}}(n(\lceil log (l(v_{max }-v_{min }))\rceil ))$ memory.


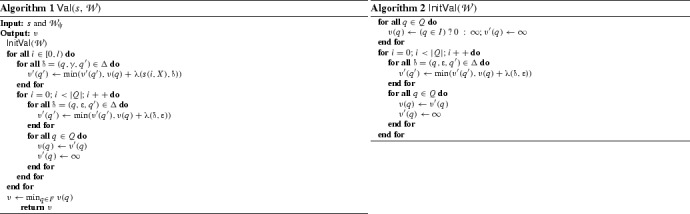


#### Example 3

**Fig. 7:**
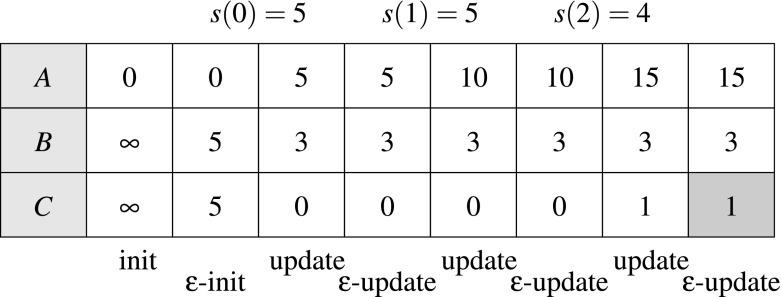
Example—computation of $v(s, {\mathcal {W}}_{\upvarphi })$

Consider the STL property $\upvarphi$ from Example 1, the associated weighted edit automaton ${\mathcal {W}}_{\upvarphi }$ from Fig. 1 and the signal^4^ $s~:~[0,2] \rightarrow [0,5]$ such that $s(0) = 5$, $s(1) = 5$ and $s(2) = 4$. It is clear that $(s,0) \not \models \upvarphi$, since $s(2) = 4$, while there was not a single $0 \le i < 2$ where $s(i) < 3$. We illustrate in Fig. 7 the computation of $v(s,{\mathcal {W}}_{\upvarphi })$. We can see that with the signal *s*, we can reach one of the accepting states (*B* or *C*) with the value 1. This value corresponds to one substitution operation, replacing the value of 4 in *s*(2) by 5, which allows vacuous satisfaction of the property $\upvarphi$.

## Implementation and case study

We now describe our implementation of quantitative monitors for STL. In order to evaluate our approach, we conducted two case studies. The first case study takes specification from automotive benchmarks published in [5]. In second case study we applied our quantitative monitors on Single Edge Nibble Transmission (SENT) protocol, a standard for sensor to controller communication in the automotive industry [24].

In both cases, parser for STL formulas is developed in Java using ANTLR [32]. In order to translate STL properties into temporal testers, we take basic temporal testers for STL operators and create their product. Then, we convert such top level temporal tester into an acceptor automaton. We use JAutomata [22] library to represent the testers and the acceptors. We then generate quantitative monitor code in Verilog HDL. The resulting monitor is a hardware implementation of the weighted automata and the underlying algorithm for computing the weighted edit distance. The monitor operates at the frequency limited by the maximum achievable frequency of the FPGA.

### Benchmarks for automotive systems

For the evaluation of our approach, we apply it to two benchmarks implemented in Matlab/Simulink and published in [5].

#### Automatic transmission system

We first consider the slightly modified automatic transmission deterministic Simulink demo provided by Mathworks as our system-under-test (SUT). It is a model of an automatic transmission controller that exhibits both continuous and discrete behavior. The system has two inputs—the throttle $u_{t}$ and the break $u_{b}$. The break allows the user to model variable load on the engine. The system has two continuous-time state variables—the speed of the engine $\upomega$ (RPM), the speed of the vehicle *v* (mph) and the active gear $g_{i}$. The system is initialized with zero vehicle and engine speed. It follows that the output trajectories depend only on the input signals $u_{t}$ and $u_{b}$, which can take any value between 0 and 100 at any point in time. The Simulink model contains 69 blocks including 2 integrators, 3 look-up tables, 2 two-dimensional look-up tables and a Stateflow chart with 2 concurrently executing finite state machines with 4 and 3 states, respectively. The benchmark defines 8 STL formalized requirements that the system shall satisfy, shown in Table 1.

**Table 1:** Automatic transmission properties [5]

	$$\upvarphi $$
$$\upvarphi _{1}$$	
$$\upvarphi _{2}$$	
$$\upvarphi _{3}$$	
$$\upvarphi _{4}$$	
$$\upvarphi _{5}$$	
$$\upvarphi _{6}$$	
$$\upvarphi _{7}$$	
$$\upvarphi _{8}$$	

**Fig. 8:**
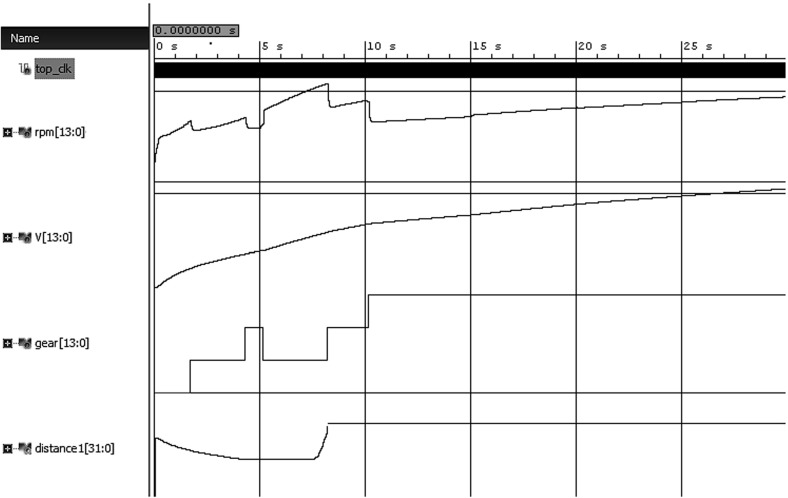
A simulation trace *s* from the automatic transmission model and $d_{W}(s, \lnot \upvarphi _{6})$

We now describe the evaluation setup. We simulated the Simulink model with fixed-step sampling and recorded the results. The obtained traces, as the one shown in Fig. 8, were then further discretized with the uniform quantization. We have obtained 751 samples from the Simulink model and normalized all variables’ value domain to the interval [0, 5000] which is the range of RPM variable, thus achieving fair reasoning about their substitution cost. We designed a testbench in Verilog to stimulate the monitor with generated values from the Simulink model. We used Xilinx Vivado to perform monitor simulation and synthesis.

Figure 8 illustrates the monitoring results for $\upvarphi _{6}$ on a specific gear input. In the depicted scenario, the speed does not reach 120 mph in 4 s, a sufficient condition for the satisfaction of the formula. In order to violate the formula, we need to alter both *v* and $\upomega$ signals such that 1) *v* reaches 120 mph at any moment within the first 4 s; and (2) $\upomega$ remains continuously below 4500 rpm. These alterations result in (1) a single substitution happening within the first 4 s which is necessary to bring *v* to 120 mph; and (2) the accumulation of substitution costs in the interval between 7 and 8 s of the simulation where $\upomega$ actually exceeds 4500 rpm. Note that the robustness degree decreases in the first 4 s. This happens because the actual *v* increases and the substitution cost needed for *v* to reach 120 mph is continuously being improved.

The evaluation results are shown in Table 2. We tested the correctness of STL to automata translation by generating both acceptors for $\upvarphi$ and $\lnot \upvarphi$. The presented robustness degrees are not normalized, which can be statically computed using the formula from Sect. 4. It is clear from our table that either the distance from $\upvarphi$ or from its negation is always 0. The dominant type of resources when implementing our monitors on FPGA hardware are LUTs. This is not surprising, due to the large combinatorial and arithmetic requirements of the computation. We can also note that the size of our monitors is sensitive to the timing bounds in the formulas and the sampling period of the input signals. Our monitor automata enumerate clock ticks instead of using a symbolic representation. The enumeration is necessary because state—clock valuation pairs can have different values associated and thus cannot be grouped. We were not able to generate monitors for $\upvarphi _{5}$ and $\upvarphi _{8}$ due to the state explosion. However, $\upvarphi _{5}$ can be decomposed into 4 independent sub-properties. We can see several ways to handle large properties such as $\upvarphi _{8}$ that we will investigate in the future—by reformulating the specification using both past and future operators, by using larger sampling periods (smaller time bounds in the formula) and by using more powerful FPGA.

**Table 2:** Evaluation results for automatic transmission benchmark

	$\uprho$	${\mathcal {W}}_{\upvarphi }$				${\mathcal {W}}_{\lnot \upvarphi }$			
		|*Q*|	$|\Delta |$	#FF	#LUT	|*Q*|	$|\Delta |$	#FF	#LUT
$\upvarphi _{1}$	$-$ 2528	2	2	62	260	4	8	94	657
$\upvarphi _{2}$	$-$ 11,423	2	2	75	306	4	11	107	799
$\upvarphi _{3}$	1000	496	1374	4106	53,033	992	2878	8127	106,937
$\upvarphi _{4}$	1000	496	692	3061	22,777	992	1445	6025	44,968
$\upvarphi _{5}$	n/a	n/a	n/a	n/a	n/a	n/a	n/a	n/a	n/a
$\upvarphi _{6}$	5337	405	813	6540	66,085	409	903	6504	73,657
$\upvarphi _{7}$	$-$ 5336	403	903	6504	73,766	405	813	6545	66,116
$\upvarphi _{8}$	n/a	n/a	n/a	n/a	n/a	n/a	n/a	n/a	n/a

#### Fault-tolerant fuel control system

The second automotive benchmark is based on fault-tolerant fuel control system model [5, 23]. This system ensures proper air-to-fuel ratio in modern car engines. It must be adaptive to any kind of external failures, such as sensor failures. Since the occurrence of failures is modeled by Poisson stochastic processes, this benchmark will evaluate our quantitative monitors with a model of a Stochastic Cyber Physical System.

The system has throttle as an input which affect failure arrival rates. The change in detected fuel level can be caused either by throttle or a sensor failure. Such change directly affects air-to-fuel ratio $\uplambda$ which is the output of the model. We sample this variable over time in order to create stimulus for our monitors. We collected 10,000 samples from the model output. We rounded double precision output to 2 decimals, and multiplied it by 100 for easier representation in hardware testbench.

The requirement for air-to-fuel ratio $\uplambda$ specifies that no matter what kind of disturbance in system occurs, the value of $\uplambda$ must stabilize within certain value limit in specified time window. We call this a bounded stabilization property and formalize it in STL with the following formula:As suggested by the authors of [5], we use $V_{limit} = 1.1 \cdot V_{id}$, where $V_{id}$ corresponds to ideal air-to-fuel ratio when no throttle change or a sensor failure occurs.

**Fig. 9:**
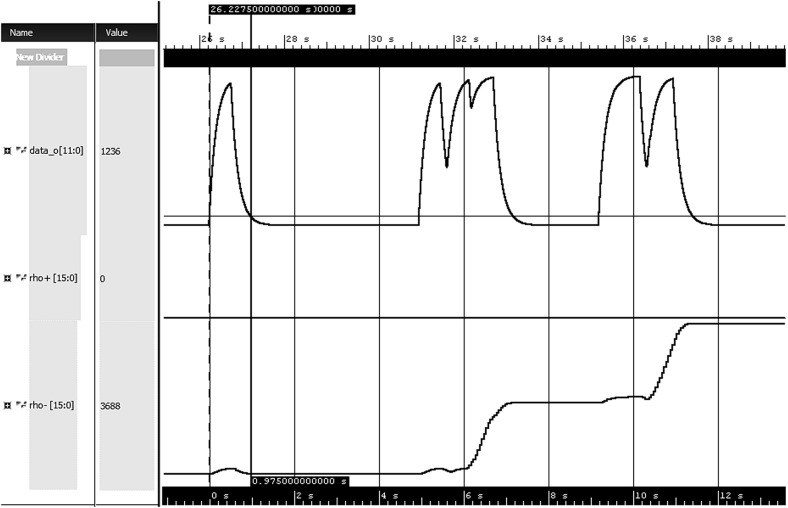
Calculated positive and negative robustness for obtained air-to-fuel $\uplambda$

In Fig. 9 we can observe change of $\uplambda$ and robustness values w.r.t. the formula. We see several $\uplambda$ pulses caused by the disturbance in the system. Due to the initial conditions, negative robustness is greater than zero. The first pulse is satisfying the requirement since it stabilizes to required $V_{limit}$ within 1 second time window. Since it satisfies the bounded stabilization property and does not add any WED cost, the negative robustness value remains the same before and after the pulse.

The next disturbance in the system generates more impact on air-to-fuel ratio. In this case the signal does not stabilize fast enough. Therefore the WED algorithm suggests to substitute problematic parts of the trace with correct values. Since the substitution costs accumulate, the negative robustness keeps increasing. Positive robustness equals zero throughout the simulation due to the fact that the trace is violating the formula from the start.

We report on monitor size and FPGA resource consumption in Table 3. The large negative robustness value is due to several bounded stability violations and significantly larger number of samples compared to automatic transmission system testbench. Increased resource consumption is consequence of the fact that the formula uses future time STL operators over bounded time intervals.

**Table 3:** Evaluation results for fault-tolerant fuel control system properties [5]

	$\uprho$	${\mathcal {W}}_{\upvarphi }$				${\mathcal {W}}_{\lnot \upvarphi }$			
		|*Q*|	$|\Delta |$	#FF	#LUT	|*Q*|	$|\Delta |$	#FF	#LUT
$\upvarphi _{9}$	$-$ 43,878	882	1493	13,203	119,989	1574	2648	23,624	212,341

### SENT protocol case study

**Fig. 10:**
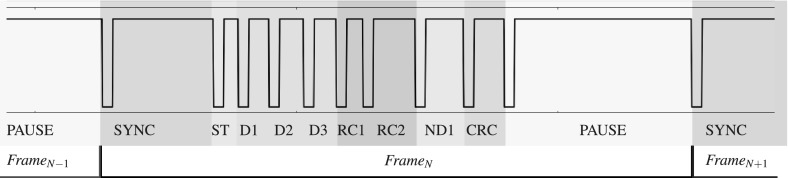
A SENT frame starts with a mandatory synchronization pulse (SYNC), followed by a status nibble (ST), data nibbles (D1, D2, D3), rolling counters (RC1, RC2), bit inverse of D1 (ND1), cyclic redundancy check (CRC), and finishes with an optional pause

Single Edge Nibble Transmission Protocol (SENT) protocol is an industry standard SAE J2716 [24] that specifies unidirectional data encoding scheme from transmitting device (typically a sensor) to a controller. It is usually found in automotive applications such as the Electronic Power Steering (EPS), or the Electronic Braking System (EBS) where sensors continuously send data to the Engine Control Unit (ECU). SENT information is encoded into frames and transmitted over a single line in serial fashion. A SENT frame consists of several consecutive components, each defined by a dedicated pulse. Presence of certain pulses, such as the pause pulse, may vary depending on the system configuration. Data is always transmitted in nibbles and encoded in data nibble pulse length (Pulse Width Modulation), regardless of the configuration. Figure 10 shows an example of a SENT frame.

#### Formalized SENT requirements

**Fig. 11:**
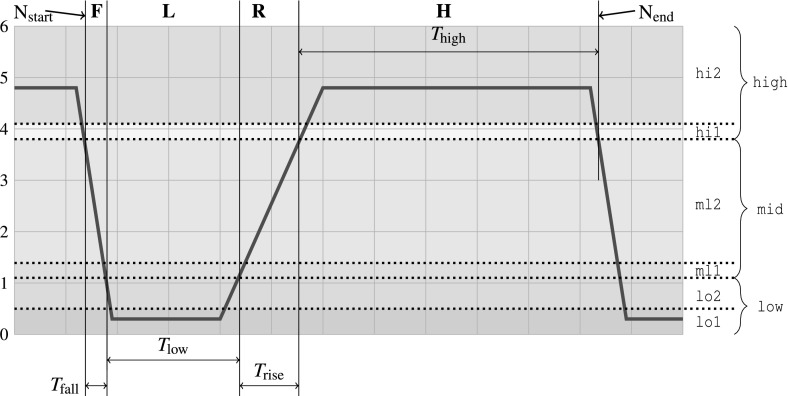
SENT nibble pulse: a pulse starts ($N_{\text {start}}$) with a falling edge f, followed by a low region l, followed by a rising edge r, followed by a high region h

In order to communicate without errors, SENT transmitter must comply to timing and electrical requirements specified by the standard. We focus on monitoring timing requirements of the rising and falling edges of a pulse. If these timing constraints are not met, it is not guaranteed that the controller will be able to decode the data from the pulse. A correct SENT pulse with timing requirements is shown in Fig. 11.

The timing requirements of interest can be stated in natural language as follows: the fall/rise time from $V_1$ to $V_2$ must be no longer than $T_{fall}$/$T_{rise}~\upmu \hbox {s}$. Before applying our approach we formalize the requirements with following STL formulas:where *start* and *end* operators are defined by  and , respectively. The SENT standard allows the following values: $T_{fall} \le 6.5~\upmu \hbox {s}$ and $T_{rise} \le 18~\upmu \hbox {s}$. Voltage levels are also specified in the standard, however in our experiments they are scaled to the Analog-to-Digital converter output range.

#### Evaluation results

In order to test the monitors with realistic data, we recorded output from a real magnetic sensor which implements the SENT protocol. We used the Hall-effect sensor with SENT interface from Infineon Technologies. The Hall-effect cell in this sensor measures the magnetic flux. Such information can be used for linear and angular position sensing. In the automotive domain, this sensor is used to sense steering torque and pedal and throttle position.

According to the SENT standard, devices are configured prior to operation. Therefore, we are allowed to assume that the configuration of SENT frame is static and its structure cannot change during runtime.

**Fig. 12:**
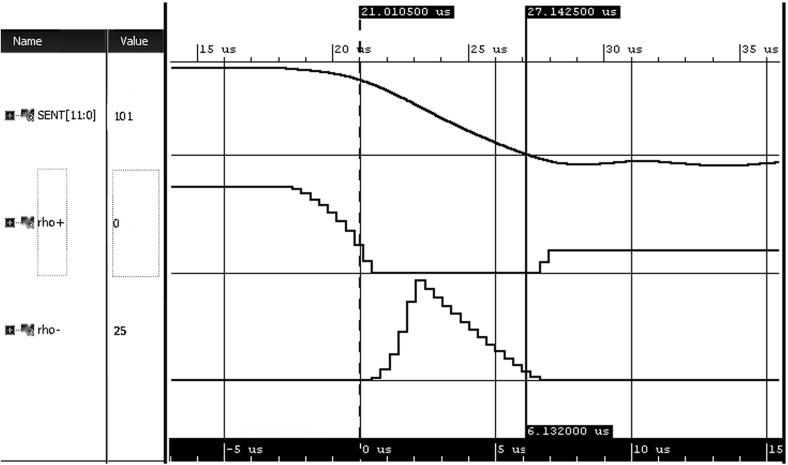
Calculated positive and negative robustness for SENT pulse falling edge which satisfies $T_{fall}$ requirement

In Fig. 12 we can see the first SENT requirement monitored on a trace which represents a correct SENT pulse falling edge. For this pulse we compute both positive and negative robustness degree. In the beginning of the trace, the left hand side of the implication is not satisfied, therefore the entire formula is trivially satisfied and the negative robustness is zero. In contrast, the positive robustness is equal to the WED cost of creating a violating trace—which can be done simply by substituting $$high $$ sample with $$mid $$, thus making $$\downarrow high $$ condition true and the entire formula false. We note that the positive robustness decreases in the course of the execution—this happens because the robustness algorithm dynamically discovers a cheaper way to transform the trace into a violating one.

We now analyze these results in more detail. At the moment when the left hand side of the implication becomes satisfied (dashed yellow marker in Fig. 12, the right hand side of the formula is not yet satisfied. This results in an increase of the negative robustness that comes from the accumulated WED substitution costs needed to disarm the $\downarrow high$ condition. After observing a sufficient number of trace samples, the robustness algorithm realizes that it is cheaper to perform substitutions at the low value end of the falling edge in order to make the right hand side of the formula hold. As a consequence, the negative robustness starts also decreasing. Finally, the monitor starts observing the samples that satisfy the right hand side of the implication, thus also satisfying the entire formula. This results in the negative robustness dropping to zero, but also in an increase of the positive robustness (see the trace segment after the yellow mark in Fig. 12). The small positive robustness degree conveys two important messages: (1) the observed execution satisfies the timing requirements; and (2) a small change of the trace could violate the requirement.

**Fig. 13:**
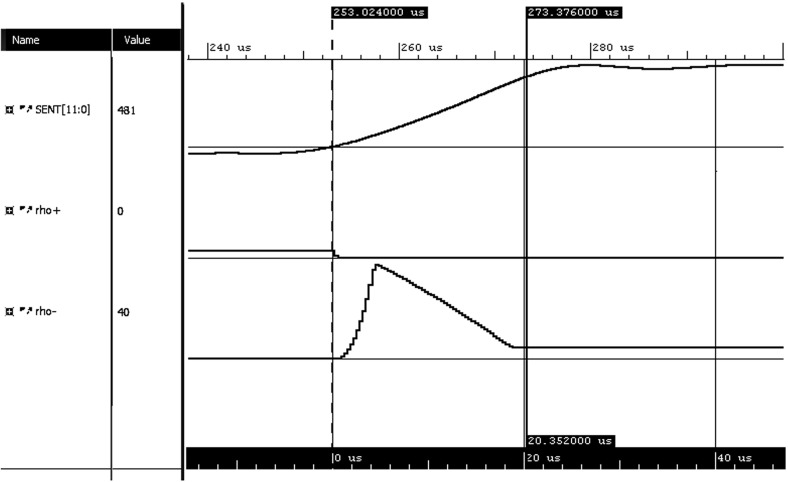
Calculated positive and negative robustness for SENT pulse rising edge which violates $T_{rise}$ requirement

In Fig. 13 we can see the rising edge timing requirement monitored on a trace which represents a violating SENT pulse. The violating pulse was artificially created from a correct trace which was recorded from the actual sensor. The violation was created by replaying the correct recorded values at a slower speed, which prolonged the rising edge length.

In this case the evolution of positive and negative robustness degree over time is converse to the previous case. The obvious difference is that the final value of the positive robustness is zero and the negative robustness degree is non-negative. This is valid result because the trace is violating the rising edge timing requirement $T_{rise} \le 18~\upmu \hbox {s}$. The negative robustness is larger than the positive robustness degree of the previous example in Fig. 12, due to the larger cost of compensating for timing violation of the rising edge.

In Table 4 we report on FPGA resource consumption of the generated monitors. Flip-flops (FF) represent memory elements which are used to implement automaton states. Lookup tables (LUT) implement dynamic programming calculations on taking the minimum cost in every step for every state. The linear dependency between increase in number of transitions of automaton and amount of LUTs used can be observed in Table 4. The same conclusion can be drawn for the number of states and the number of FFs consumed.

**Table 4:** Evaluation results for SENT protocol properties

	$\uprho$	${\mathcal {W}}_{\upvarphi }$				${\mathcal {W}}_{\lnot \upvarphi }$				SAT
		|*Q*|	$|\Delta |$	#FF	#LUT	|*Q*|	$|\Delta |$	#FF	#LUT	trace
$\upvarphi _{10}$	11	208	627	3272	50,046	498	1945	7745	148,852	Yes
$\upvarphi _{11}$	$-$ 41	558	1677	8865	136,321	1338	5224	21,191	405,604	No

## Conclusions and future work

In this paper, we proposed a new procedure for measuring robustness of STL properties based on the weighted edit distance. The distance is cumulative by definition which allows robustness degree to be sensitive on the number of violations of the formula. It is also sensitive to the length of the signal, but also to the sampling rate and the number of components in the signal. Distance normalization would help to obtain a uniform measure of “goodness” of a behavior. Although in this paper we focus on the quantitative semantics of STL, the weighted edit distance can be applied to other specification languages over finite signals.

Our FPGA implementation provides the possibility to quantify the distance to the violation of safety requirements in real-time on actual or emulated hardware. We have successfully demonstrated our approach to check relevant safety properties in the automotive domain, i.e. by monitoring the behavior of the engine through the observation of essential signals such as air-to-fuel ratio. Furthermore, we show that our method is also suitable to verify well-established industrial standard such as the SENT protocol.

*Future work* Treating the value domain symbolically is natural and we exploit this fact in the paper. On the other hand, combining quantitative semantics with symbolic time is not straightforward. In the qualitative case, representing the time symbolically can be done because there is a certain equivalence between states that have the same discrete location and different clock valuations, and such states can be grouped together. In the quantitative setting, this is not the case—two states with the same discrete location and different clock valuations will in general have different values and hence cannot be grouped together. Such a symbolic representation of quantitative states might be possible if some accuracy can be dropped. We will consider extending our algorithm to automata with discrete clocks.

We plan to exploit the quantitative robustness degree of our framework to gain predictive ability and extend our monitors for the system health and fail-aware applications.

## Figures and Tables

**Fig. 14:**
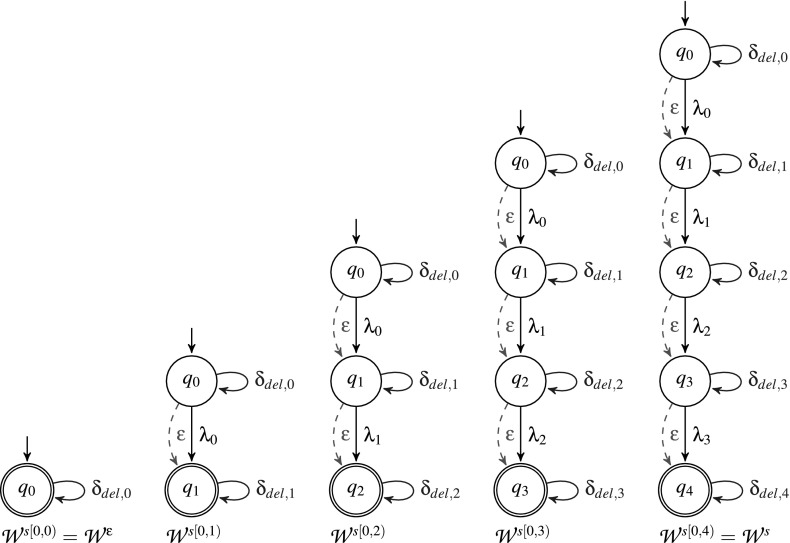
An array of SWWAs ${\mathcal {W}}^{s[0,j)}$ that model all possible edit operations on each prefix $s[0,j) \in P(s)$ of $s \in L(\upvarphi )$

## A Appendix: Theorem Proofs

### Proposition 1

The weighted edit distance is a distance.

### Proof

The proof is similar to the proof that edit distance is a distance, and is given for completeness reason.

1. $d_{W}(s,s) = 0:$ in order to transform *s* to itself, no insertions or deletions are needed, and all substitutions have cost 0.
2. $d_{W}(s_{1},s_{2}) = d_{W}(s_{2},s_{1}):$ insertions and deletions are inverses of each other, while substitution is symmetric.
3. $d_{W}(s_{1},s_{2}) \ge 0:$ by definition, all costs are greater or equal to 0.
4. $d_{W}(s_{1}, s_{2}) \le d_{W}(s_{1}, s) + d_{W}(s,s_{2}) :$ by definition, $d_{W}(s_{1},s_{2})$ is the minimum summation of insertion, deletion and substitution costs needed to transform $s_{1}$ to $s_{2}$. Transforming $s_{1}$ to *s*, and then *s* to $s_{2}$ is one way of transforming $s_{1}$ to $s_{2}$, and hence cannot be cheaper than $d_{W}(s_{1}, s_{2})$.

$$\square $$

For the next two theorems, we first prove an auxiliary lemma.

### Lemma 1

Let *s* be a signal, ${\mathcal {W}}$ a weighted symbolic automaton and $\uppi _{1}$ and $\uppi _{2}$ two paths in ${\mathcal {W}}$ induced by *s* such that both paths terminate in the same state $q \in Q$ and $v_{\uppi _{1}}(s,{\mathcal {W}}) < v_{\uppi _{2}}(s,{\mathcal {W}})$. Then, for all continuations $s'$ of *s* and all paths $\uppi _{1} \cdot \uppi$ and $\uppi _{2} \cdot \uppi$ induced by $s \cdot s'$, $v_{\uppi _{1} \cdot \uppi }(s \cdot s',{\mathcal {W}}) < v_{\uppi _{2} \cdot \uppi }(s \cdot s',{\mathcal {W}})$.

### Proof

The proof follows directly from the definition of path and the definition of path value in weighted automaton ${\mathcal {W}}$. Each continuation path of either $\uppi _{1}$ or $\uppi _{2}$ must start from the state *q*. Since both $\uppi _{1}$ and $\uppi _{2}$ terminates at state q it means that all symbols from *s* are consumed once a state $q'$ is reached. For this reason, it is clear that only $s'$ affects transition weights in $\uppi$. As a consequence, and by the definition of $v_{\uppi }(s,{\mathcal {W}})$ the following holds:

$$\begin{aligned} v_{\uppi _{i} \cdot \uppi }(s \cdot s',{\mathcal {W}}) = v_{\uppi _{i}}(s,{\mathcal {W}}) + v_{\uppi }(s', {\mathcal {W}}). \end{aligned}$$

By definition, $v_{\uppi }(s,{\mathcal {W}})$ is a sum of non-negative transition weights. Consequently, the following holds:

$$\begin{aligned} v_{\uppi _{1}}(s ,{\mathcal {W}}) + v_{\uppi }(s', {\mathcal {W}}) < v_{\uppi _{2}}(s ,{\mathcal {W}}) + v_{\uppi }(s', {\mathcal {W}}. \end{aligned}$$

$$\square $$

### Theorem 1

$$d_{W}(s,\upvarphi ) = v(s, {\mathcal {W}}_{\upvarphi })$$.

Due to the length of the proof, it is presented in separate appendix below.

### Theorem 2

Val$$(s, {\mathcal {W}}) = v(s, {\mathcal {W}})$$.

### Proof

The algorithm iteratively explores the paths in ${\mathcal {W}}$ induced by *s* and computes their values after reading the current prefix. Although the number of paths grows with the number of iterations, by Lemma 1 it is sufficient to keep in every iteration only the minimum value of reaching each state in *Q* after reading the current prefix. We also bound the number of consecutive silent transitions (insertion operations) to |*Q*| in every iteration—it is guaranteed that if a state $q'$ is reachable from state *q*, it can be reached within |*Q*| steps. When the main loop of the algorithm terminates, the values associated to each state clearly correspond to the minimal values of reaching them with the signal *s*—the minimum value of an accepting state hence corresponds to $v(s, {\mathcal {W}})$. $\square$

### Theorem 3

Given a signal *s* of length *l* defined over *X* and a weighted automaton ${\mathcal {W}}$ with *n* states and *m* transitions, $\textsc {Val}(s,{\mathcal {W}})$ takes in the order of ${\mathcal {O}}(l(nm))$ iterations to compute the value of *s* in ${\mathcal {W}}$, and requires in the order of ${\mathcal {O}}(n(\lceil log (l(v_{max }-v_{min }))\rceil ))$ memory.

### Proof

In Algorithm 1, there are *l* main iterations, and in each iterations one needs to do *m* updates due to substitutions/deletions and *mn* updates due to $\upvarepsilon$-transition propagation. For the space complexity, we need to keep for each state a value, that can be at most $l(v_{max }- v_{min })$ and can be encoded in binary. $\square$

## B Proof of Theorem 1

In this section, we provide the proof that $d(s, \upvarphi ) = v(s, {\mathcal {W}}_{\upvarphi })$. In order to achieve this goal, we decompose the problem into the following smaller instances:

where $s'$ represents an arbitrary word from the language of formula $\upvarphi$.

In (1), we first decompose ${\mathcal {W}}_{\upvarphi }$ into a (possibly infinite) union of weighted symbolic automata ${\mathcal {W}}^{s'}$, where each ${\mathcal {W}}^{s'}$ models all possible edit operations that are applicable to transform an arbitrary signal *s* into another signal $s' \in L(\upvarphi )$. In (2), we then show that finding $s' \in L(\upvarphi )$ that minimizes the distance $d_{W}(s,s')$ is equivalent to finding the $s' \in L(\upvarphi )$ that minimizes the value $v(s, {\mathcal {W}}^{s'})$. Finally, in (3) we show that for an arbitrary $s'$, the distance $d_{W}(s,s')$ equals to the value of the value $v(s,{\mathcal {W}}^{s'})$. We proceed in the bottom up fashion, by proving first (3), i.e. $d(s, s') = v(s, {\mathcal {W}}^{s'})$, where ${\mathcal {W}}^{s'}$ is a weighted edit automaton obtained by applying the procedure from Sect. 5.1.2 to the automaton ${\mathcal {A}}^{s'}$ that accepts only the trace $s'$.

### Definition 5

Given a signal *s*, we define a *single word acceptor* (SWA) for *s*, ${\mathcal {A}}^s$, as the minimal automaton such that $L({\mathcal {A}}^s) = \{s\}$ holds. We denote by ${\mathcal {W}}^{s}$ the *single word weighted acceptor* (SWWA) that is constructed from ${\mathcal {A}}^{s}$ by applying the procedure from Sect. 5.1.2.

We note that for every *s* of size *n*, both ${\mathcal {A}}^{s}$ and ${\mathcal {W}}^{s}$ consist of a sequence of locations $q_{0},\ldots , q_{n}$, where $q_{0}$ is the only initial location, $q_{n}$ is the only final location, and for all $0 < i \le n$, the incoming transitions to $q_{i}$ have a source either in $q_{i-1}$ or in $q_{i}$. We define by *P*(*s*) the set of all prefixes of a signal *s*, where *s*[0, *i*) denotes the prefix of *s* of size *i*, where $0 \le i \le |s|$. In Fig. 14, we illustrate the array of weighted edit automata ${\mathcal {W}}^{s[0,i)}$ for the prefixes of the signal *s* of size 4.

### Lemma 1

Let *s* and $s'$ be two arbitrary signals of size *m* and *n*, respectively. Then, for all $0 \le i \le m$ and $0 \le j \le n$, we have that $d_W(s[0,i),s'[0,j)) = v(s[0,i), {\mathcal {W}}^{s'[0,j)})$.

### Proof

We prove this lemma by induction.

*Base case:* We first prove that (1) $d_W(\upvarepsilon , s'[0,j)) = v(\upvarepsilon , {\mathcal {W}}^{s'[0,j)})$ for all $0 \le j \le n$ and (2) $d_W(s[0,i), \upvarepsilon ) = v(s[0,i), {\mathcal {W}}^{\upvarepsilon })$ for all $0 \le i \le m$. In the case (1), by the definition of the weighted edit distance $d_W(\upvarepsilon , s'[0,j)) = jc_{i}$. The cheapest path from the initial to the finite location in ${\mathcal {W}}^{s'[0,j)}$ induced by an empty word is by taking *j* consecutive $\upvarepsilon$ (insertion) transition, each inducing a cost of $c_i$. In the case (2), by the definition of the weighted edit distance $d_W(s[0,i), \upvarepsilon ) = ic_{d}$. The cheapest path from the initial to the accepting state in ${\mathcal {W}}^{\upvarepsilon }$ induced by *s*[0, *i*) is to consume the *i* letters by consecutive self-loop (deletion) transition, each inducing a cost of $c_d$.

*Inductive step:* By inductive hypothesis, we assume that $d(s[0,i-1),s'[0,j-1)) = v(s[0,i-1), {\mathcal {W}}^{s'[0,j-1)})$, $d(s[0,i-1),s'[0,j)) = v(s[0,i-1), {\mathcal {W}}^{s'[0,j)})$ and $d(s[0,i),s'[0,j-1)) = v(s[0,i), {\mathcal {W}}^{s'[0,j-1)})$. We now prove that $d(s[0,i),s'[0,j)) = v(s[0,i), {\mathcal {W}}^{s'[0,j)})$. By definition of the weighted edit distance, we have that

We now prove that

We first observe that $${\mathcal {W}}^{s'[0,j)}$$ has only one final location $$q_j$$. By the definition of the weighted symbolic automata, path values are non-negative and additive, and by the definition of $${\mathcal {W}}^{s'[0,j)}$$, any location $$q_{j'}$$, where $$0 < j' \le j$$ can be reached in one step only from $$q_{j'}$$ or $$q_{j' -1}$$. It follows that it is sufficient to consider *s*[0, *i*) and $$s[0,i-1)$$ and $$q_{j}$$ and $$q_{j-1}$$ in order to prove $$d(s[0,i),s'[0,j)) = v(s[0,i), {\mathcal {W}}^{s'[0,j)})$$. Let $$\uppi = \uppi ' \cdot \updelta \cdot q_j$$ be the path with minimum value induced by *s*[0, *i*) in $${\mathcal {W}}^{s'[0,j)}$$. By the the definition of $${\mathcal {W}}^{s'[0,j)}$$, $$q_j$$ has 3 incoming transitions: (1) a substitution transition from $$q_{j-1}$$ to $$q_{j}$$; (2) an $$\upvarepsilon $$ (insertion) transition from $$q_{j-1}$$ to $$q_j$$; and (3) a self-loop (deletion) transition in from $$q_j$$ to $$q_j$$.

By the definition of the value in a weighted symbolic automaton, the value of $$\uppi $$ corresponds to the value of $$\uppi '$$ to which the cost of the last transition $$\updelta $$ is added, which is the minimum of the above three cases. In the case (1), $$\uppi '$$ reaches $$q_{j-1}$$ with $$s[0,i-1)$$ consumed. The value accumulated by $$\uppi '$$ corresponds to the value of $$\uppi '$$ induced by $$s[0,i-1)$$ in $${\mathcal {W}}^{s'[0,j-1)}$$, i.e. $$v(s[0,i-1),{\mathcal {W}}^{s'[0,j-1)})$$. The added cost of the last transition corresponds to $$c_{s}(s(i), s'(j))$$. In the case (2), $$\uppi '$$ reaches $$q_{j-1}$$ with *s*[0, *i*) consumed. The value accumulated by $$\uppi '$$ corresponds to the value of $$\uppi '$$ induced by *s*[0, *i*) in $${\mathcal {W}}^{s'[0,j-1)}$$, i.e. $$v(s[0,i),{\mathcal {W}}^{s'[0,j-1)})$$. The added cost of the last transition corresponds to the cost $$c_i$$ of an insertion. In the case (3), $$\uppi '$$ reaches $$q_{j}$$ with $$s[0,i-1)$$ consumed. The value accumulated by $$\uppi '$$ corresponds to the value of $$\uppi '$$ induced by $$s[0,i-1)$$ in $${\mathcal {W}}^{s'[0,j)}$$, i.e. $$v(s[0,i-1),{\mathcal {W}}^{s'[0,j)})$$. The added cost of the last transition corresponds to the cost $$c_d$$ of a deletion. $$\square $$

### Corollary 1

$$d_W(s,s') = v(s, {\mathcal {W}}^{s'})$$

Corollary 1 is a special case of Lemma 1, where $i=m$ and $j=n$. We can now generalize Corollary 1 with the following lemma, in order to take into account all the (possibly infinite number of) signals that are in a language of an STL formula $\upvarphi$.

### Lemma 2

$$\min _{s' \in L(\upvarphi )} d_W(s,s') = \min _{s' \in L(\upvarphi )}v(s, {\mathcal {W}}^{s'})$$.

### Proof

Follows directly from Corollary 1 and the definition of a minimum. $\square$

### Corollary 2

$$d_W(s,\upvarphi ) = v(s, \bigcup _{s' \in L(\upvarphi )} {\mathcal {W}}^{s'})$$

The above corollary follows from the definition of the weighted edit distance, and the fact that $\min$ distributes over the union. We finally need to show that the decomposition of ${\mathcal {W}}_{\upvarphi }$ into $\bigcup _{s' \in L(\upvarphi )}{\mathcal {W}}^{s'}$ preserves the value induced by *s*. We first show that the above decomposition preserves paths, that is all possible sequences of edit operations that transform *s* into any $s' \in L(\upvarphi )$ and the values of these paths.

### Lemma 3

Consider an arbitrary trace *s* and an STL formula $\upvarphi$. For every path $\uppi$ in ${\mathcal {W}}_{\upvarphi }$ induced by *s*, there exists $\uppi '$ in $\bigcup _{s' \in L(\upvarphi )} {\mathcal {W}}^{s'}$ induced by *s*, and for every path $\uppi '$ in $\bigcup _{s' \in L(\upvarphi )} {\mathcal {W}}^{s'}$ induced by *s*, there exists a path $\uppi$ in ${\mathcal {W}}_{\upvarphi }$ induced by *s*, such that $v(s,\uppi , {\mathcal {W}}_{\upvarphi }) = v_{\uppi '}(s, \bigcup _{s' \in L(\upvarphi )} {\mathcal {W}}^{s'})$.

### Proof

We create a SWWA ${\mathcal {W}}^{s'}$ for every trace $s' \in L(\upvarphi )$. It follows that $\bigcup _{s' \in L(\upvarphi )} {\mathcal {W}}^{s'}$ contains the paths that model all the edit operations that transform an arbitrary *s* into an arbitrary $s' \in L(\upvarphi )$. By the construction in Sect. 5.1.2, ${\mathcal {W}}_{\upvarphi }$ also models all the edit operations that transform an arbitrary *s* into an arbitrary $s' \in L(\upvarphi )$. The substitution operations are by definition preserved in the decomposition of ${\mathcal {W}}_{\upvarphi }$ into $\bigcup _{s' \in L(\upvarphi )} {\mathcal {W}}^{s'}$, while the insertion and deletion transitions are systematically added in both ${\mathcal {W}}_{\upvarphi }$ and $\bigcup _{s' \in L(\upvarphi )} {\mathcal {W}}^{s'}$ with the same costs. It follows that for an arbitrary *s*, both ${\mathcal {W}}_{\upvarphi }$ and $\bigcup _{s' \in L(\upvarphi )} {\mathcal {W}}^{s'}$ contain paths $\uppi$ and $\uppi '$ induced by *s* that model the same edit operations with the same value. $\square$

### Lemma 4

$$v(s,{\mathcal {W}}_{\upvarphi }) = v(s, \bigcup _{s' \in L(\upvarphi )}{\mathcal {W}}^{s'})$$.

### Proof

Follows directly from the preservation of all paths and their values proved in Lemma 3 and the definition of a value in a weighted symbolic automata. $\square$

The combination of Lemma 4, Corollary 2, Lemma 2 and Corollary 1 constitutes the proof of Theorem 1.

## Footnotes

1 We use *s*(*t*) to denote the valuation vector of the variables in *X* at time *t*.

2 Although this segment of $\textsc {STL}$ is expressively equivalent to $\textsc {LTL}$, use the $\textsc {STL}$ name to highlight the explicit notions of real-time and quantitative values in the language.

3 The time in ${\mathcal {A}}_{\upvarphi }$ cannot be treated symbolically with digital clocks since every pair of states and clock valuation may behave differently with respect to the WED.

4 Since *s* has only one component, we skip the variable name.
